# Euiin-Tang Attenuates Obesity-Induced Asthma by Resolving Metaflammation

**DOI:** 10.3390/ph17070853

**Published:** 2024-06-28

**Authors:** Ye-Eul Lee, Dong-Soon Im

**Affiliations:** Department of Basic Pharmaceutical Sciences, Graduate School, Kyung Hee University, Seoul 02447, Republic of Korea; eul08@naver.com

**Keywords:** euiin-tang, obese, asthma, lung, *Coix lacryma-jobi*

## Abstract

Euiin-tang reduces obesity and hypertension. Patients with obesity may develop obesity-induced asthma (OIA) owing to phlegm dampness. This study aimed to determine whether euiin-tang alleviates high-fat diet (HFD)-induced OIA in C57BL/6 mice. OIA was developed by HFD feeding for 15 weeks in C57BL/6 mice, and euiin-tang (5 mg/10 g/day) was orally administered for the last five weeks. Oral administration of euiin-tang suppressed HFD-induced changes in body weight, liver weight, airway hypersensitivity (AHR), and immune cell infiltration in bronchoalveolar lavage fluid. Histological analysis revealed that euiin-tang treatment suppressed HFD-induced mucosal inflammation, hypersecretion, and fibrosis. The lungs and gonadal white adipose tissue showed increased expression of inflammatory cytokines (IL-1β, IL-17A, TNF-α, IL-6, IL-13, IFN-γ, MPO, and CCL2) following HFD, whereas euiin-tang inhibited this increase. HFD also increased the number of pro-inflammatory CD86^+^ M1 macrophages and decreased the number of anti-inflammatory CD206^+^ M2 macrophages in the lungs, whereas euiin-tang treatment reversed these effects. HFD induced a decrease in adiponectin and an increase in leptin, which was reversed by euiin-tang. Therefore, euiin-tang may be a potential therapeutic agent for OIA because it suppresses metaflammation as demonstrated in the present study.

## 1. Introduction

There is an alarmingly high number of patients suffering from obesity, including 650 million adults [1]. In addition, patients with obesity and asthma increasingly suffer from uncontrolled, severe respiratory symptoms [2]. Patients with obesity and asthma respond less to conventional anti-asthmatics such as corticosteroids than patients with asthma only, probably because the pathophysiology of the disease is different [3]. The pathophysiology of obesity-induced asthma (OIA) has been associated with multiple mechanisms, including chronic low-grade inflammation of adipose tissue (metaflammation) [4], NLRP3-associated M1 macrophage polarization [5], increased circulating pro-inflammatory cytokines such as IL-6, IL-8, and tumor necrosis factor (TNF)-α [6,7,8], and changes in adipokine levels (increased leptin and decreased adiponectin) [6,7,8]. The traditional use of herbal medicines has long been a valuable resource for the treatment of many health problems, including obesity. Because herbal medicines have multi-targeted mechanisms of action and relatively few side effects [9], in-depth studies are needed to identify promising anti-obesity herbal medicines for the treatment of OIA and to understand their pathophysiology.

In Korea, euiin-tang (yiyiren-tang in Chinese; yokuininto in Japanese) has been used as a herbal concoction for obesity [10,11]. Euiin-tang consists of seven herbs: semen of *Coix lacryma-jobi* L.; radix of *Angelica gigantis* Nakai; rhizoma of *Atractylodes japonica* Koidz. ex Kitam.; *Ephedra sinica* Stapf; cortex of *Cinnamomum verum* J. Presl; *Paeonia lactiflora* Pall.; and *Glycyrrhiza uralensis* Fisch. Euiin-tang has been shown to reduce weight gain associated with a high-fat diet (HFD) and decrease leptin levels [12]. In mice with excessive weight gain induced by HFD, euiin-tang granules showed anti-obesity and anti-inflammatory effects [13]. Hence, in this study, we investigated whether euiin-tang treatment reduced obesity and OIA in HFD-induced obese mice.

## 2. Results

### 2.1. Euiin-Tang Suppressed HFD-Induced Obesity in C57BL/6 Mice

**Figure 1:**
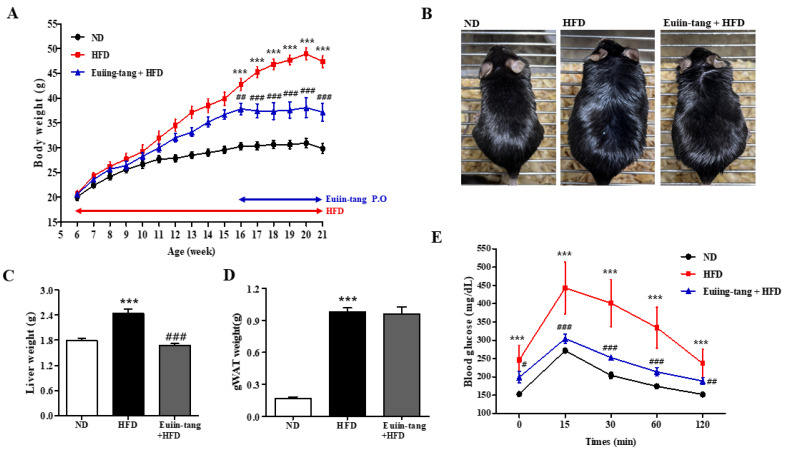
Euiin-tang suppressed HFD-induced body weight gain, liver weight gain, and glucose intolerance in mice. We evaluated the effects of euiin-tang on HFD-induced body weight gain, liver weight gain, and glucose intolerance in C57BL/6 mice: (**A**) Body weight changes. Mice were fed an HFD from 6 weeks of age for 15 weeks and orally administered euiin-tang at a dose of 5 mg/10 g of body weight for the last 5 weeks of the study period. We euthanized the mice. (**B**) Macroscopic appearance of mice. (**C**) Liver tissue weight was measured. (**D**) Gonadal white adipose tissue weight was measured. (**E**) A glucose tolerance test was conducted at 21 weeks of age. We present values as the mean ± SEM of six mice. *** *p*  <  0.001 vs. the ND group, ### *p* <  0.001, # *p*  <  0.05 and ## *p*  <  0.01 vs. the HFD group.

A model of HFD-induced OIA was developed in C57BL/6 mice [14]. From the age of 6 weeks, male C57BL/6 mice were fed either ND or HFD for 15 weeks (Figure 1A). For the last five weeks, the HFD plus euiin-tang group was administered one dose of euiin-tang (5 mg/10 g of body weight, orally) per day (Figure 1A). Compared to the ND group, HFD induced an increase in body weight by 59%, whereas euiin-tang treatment inhibited weight gain by 34% (Figure 1A,B). The HFD induced a 36% increase in liver weight, which was suppressed by administering euiin-tang (Figure 1C). HFD also induced an increase in adipose tissue weight in the gonadal region by 479% (Figure 1D), but this effect was not suppressed by euiin-tang administration (Figure 1D). During the last week of the 15th week, a glucose tolerance test was conducted to confirm obesity-induced glucose intolerance. Plasma glucose levels were higher in HFD-fed mice than in the ND-fed mice (Figure 1E). The dose of euiin-tang administered to mice improved their ability to tolerate glucose levels within the normal range (Figure 1E). Euiin-tang reduced body weight gain, liver weight gain, and glucose intolerance, but not adiposity associated with HFD consumption.

### 2.2. Euiin-Tang Suppressed AHR and Immune Cell Infiltration in BALF of C57BL/6 Mice

**Figure 2:**
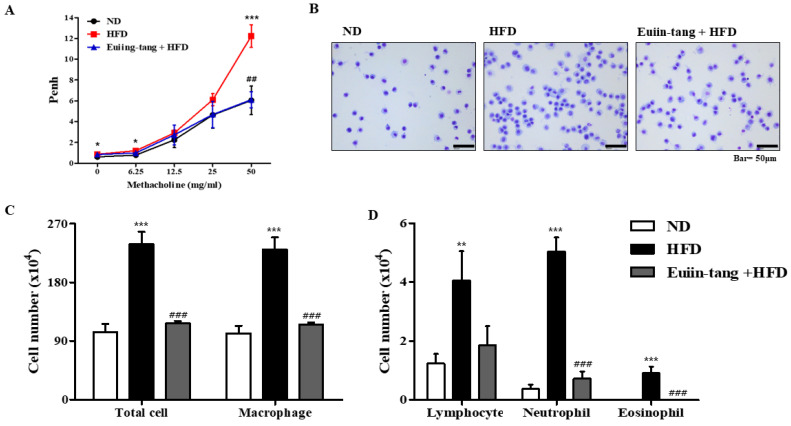
Euiin-tang suppressed HFD-induced airway hyperresponsiveness (AHR) and immune cell infiltration in the BALF of mice: (**A**) Using non-invasive whole-body plethysmography, we measured effects of HFD and euiin-tang on AHR. (**B**) Using May–Grünwald solution, we stained cells in the BALF and counted. (**C**) Total cell counts and macrophage counts in BALF. (**D**) Lymphocyte, neutrophil, and eosinophil counts in the BALF. We present values as the mean ± SEM of six mice. *** *p*  <  0.001, * *p*  <  0.05, ** *p*  <  0.01 vs. the ND group, ### *p*  <  0.001 and ## *p*  <  0.01 vs. the HFD group.

Penh was used to determine how euiin-tang affected AHR. The Penh values of obese mice were significantly higher than those of control mice at a dose of 50 mg/mL methacholine, indicating the presence of OIA (Figure 2A). Treatment with euiin-tang suppressed methacholine-induced Penh values at a dose of 50 mg/mL (Figure 2A). BALF immune cells were analyzed (Figure 2B,C), and the results showed that HFD significantly increased the total number of cells, whereas euiin-tang treatment significantly suppressed HFD-induced immune cell infiltration. Figure 2C,D shows that macrophages, lymphocytes, and neutrophils were the predominant immune cells, and eosinophils were the minority immune cells (Figure 2D), indicating that obese airway inflammation differs from allergic airway inflammation [15,16]. An increase in the number of macrophages, lymphocytes, neutrophils, and eosinophils as a result of HFD was significantly suppressed by euiin-tang treatment (Figure 2C,D).

### 2.3. Euiin-Tang Suppressed Pathological Changes in the Lungs of C57BL/6 Mice

**Figure 3:**
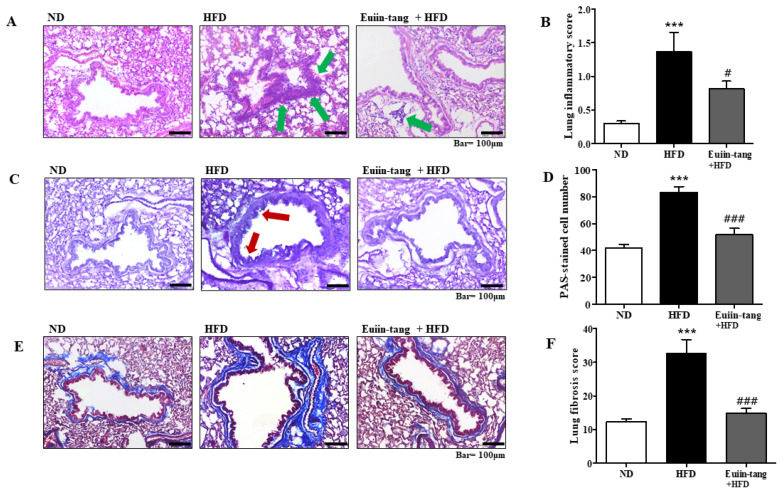
Euiin-tang suppressed HFD-induced histological changes in the lungs of mice: (**A**) Panels show H&E-stained sections of lung tissues from the ND, HFD, and euiin-tang-treated HFD groups. Small navy-blue dots around the bronchioles indicate neutrophils. Neutrophils were rarely observed in the ND group, whereas they accumulated densely around the bronchioles in the HFD group (indicated by green arrows). However, neutrophil accumulation was less notable in the HFD + euiin-tang group than in the HFD group. (**B**) Histogram of inflammation scores. (**C**) Panels show PAS/hematoxylin-stained sections of lung tissues from the ND, HFD, and euiin-tang-treated HFD groups. In PAS staining, mucin is stained purple. In the HFD group, a darker and denser purple color is observed surrounding the bronchiole compared to that in the ND group. However, mucin staining in the euiin-tang-treated group was lighter and thinner than that in the HFD group. (**D**) Histogram of PAS-stained cells. (**E**) Panels show MT-stained sections of lung tissues from the ND, HFD, and euiin-tang-treated HFD groups. (**F**) Histogram of fibrous areas. We present values as the mean ± SEM of six mice. *** *p*  <  0.001 vs. the ND group, ### *p*  <  0.001 and # *p*  <  0.05 vs. the HFD group.

Histopathological analyses were performed to determine the extent of obesity-induced airway inflammation. HFD-treated mice showed immune cell accumulation in the lungs, whereas euiin-tang treatment reduced this accumulation in H&E-stained lung sections (Figure 3A). A subjective scale ranging from 0 to 3 was used to assess lung inflammation. The average inflammation score was approximately 1.4 in the HFD group, and when euiin-tang treatment was applied, the inflammation score significantly decreased to the same degree as in the ND group (Figure 3B). HFD induced an increase in mucus secretion in the airways, whereas euiin-tang suppressed it (Figure 3C). Lung samples from HFD-fed mice revealed PAS-positive goblet cells surrounding the bronchial airways (Figure 3C), indicating that mucin production increased and the goblet cells became hyperplastic. In the HFD group, the bronchioles were surrounded by dense dark purple layers, which became thinner and less intense after euiin-tang treatment (Figure 3C). There were approximately 40 PAS-positive cells/mm in the ND group, and approximately 80 PAS-positive cells/mm in the HFD group (Figure 3D). The number of PAS-positive cells was significantly reduced after treatment with euiin-tang (Figure 3D). A significant increase in blue-stained fibrotic areas was observed in MT-stained sections when the HFD was applied, whereas euiin-tang significantly reduced the fibrotic areas (Figure 3E,F).

### 2.4. Euiin-Tang Suppressed Inflammatory Cytokine Levels in the Lungs and Gonadal White Adipose Tissue in C57BL/6 Mice

**Figure 4:**
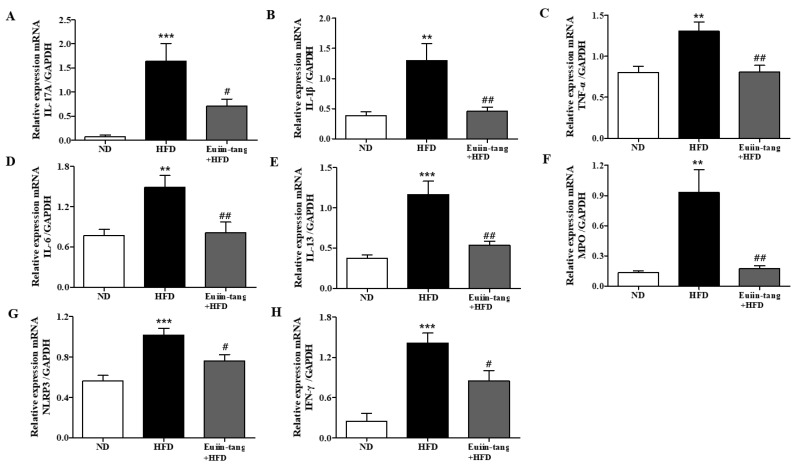
Euiin-tang suppressed the HFD-induced increase in the inflammatory response in the lungs of mice: (**A**–**H**) mRNA expression of IL-17A (**A**), IL-1β (**B**), TNF-α (**C**), IL-6 (**D**), IL-13 (**E**), MPO (**F**), NLRP3 (**G**), and IFN-γ (**H**) in mouse lungs. The levels of cytokine mRNAs are presented as relative expression to GAPDH mRNA levels. We present values as the means ± SEMs (*n* = 6) *** *p* < 0.001 and ** *p* < 0.01 vs. the ND group, ## *p* < 0.01, # *p* < 0.05 vs. the HFD-treated group.

Owing to the presence of immune cells in the lungs, several inflammation-related genes have been identified. These included IL-17A, IL-1β, TNF-α, IL-6, IL-13, MPO, NLRP3, and INF-γ [15,17]. Using qRT-PCR, we monitored the expression of pro-inflammatory Th17 (IL-17a), Th1 (IFN-γ, TNF-α, and IL-6), Th2 (IL-13), inflammasome-related genes (IL-1β and NLRP3), and a neutrophil marker gene (MPO) in the lungs. A significant increase in pro-inflammatory gene expression was observed in the HFD group, which was significantly suppressed by euiin-tang (Figure 4).

**Figure 5:**
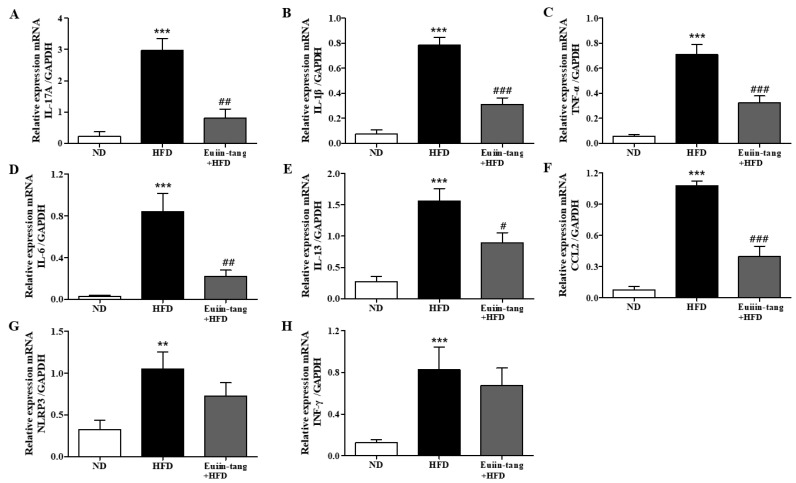
Euiin-tang suppressed the HFD-induced increase in the inflammatory response in the gonadal adipose tissues of mice: (**A**–**H**) mRNA expression of IL-17A (**A**), IL-1β (**B**), TNF-α (**C**), IL-6 (**D**), IL-13 (**E**), CCL2 (**F**) NLRP3 (**G**), and IFN-γ (**H**) in the gonadal white adipose tissue. The levels of cytokine mRNAs are presented as relative expression to GAPDH mRNA levels. We present values as the means ± SEMs (n = 6) *** *p* < 0.001 and ** *p* < 0.01 vs. the ND group, ### *p* < 0.001, # *p* < 0.05 and ## *p* < 0.01 vs. the HFD-treated group.

Because chronic low-grade systemic inflammation (metaflammation) in the gonadal adipose tissue is associated with airway inflammation in OIA [18], we examined the levels of inflammatory cytokines in the gonadal white adipose tissue using qRT-PCR. A significant increase in the levels of IL-17A, IL-1β, TNF-α, IL-6, IL-13, CCL2, NLRP3, and INF-γ was observed in the adipose tissues of HFD group mice (Figure 5). Euiin-tang treatment significantly inhibited this increase (Figure 5).

### 2.5. Euiin-Tang Suppressed ILCs and Increased Anti-Inflammatory M2 Macrophages in the Lungs of Mice

**Figure 6:**
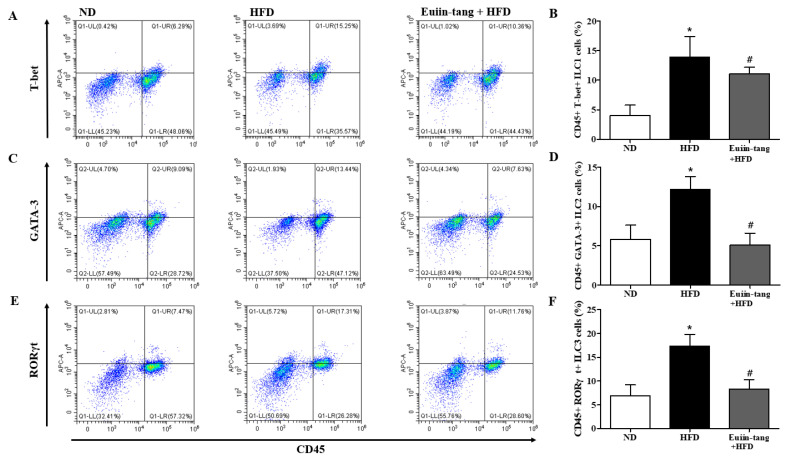
Euiin-tang suppressed the HFD-induced increase in ILCs in the lungs: Representative flow cytometry dot plots of FACS analysis of CD45^+^FcεR1^−^T-bet^+^ ILC1 (**A**), CD45^+^FcεR1^−^GATA-3^+^ ILC2 (**C**), and CD45^+^FcεR1^−^RORγt^+^ ILC3 cells (**E**) in the lungs. Percentage of CD45^+^FcεR1^−^T-bet^+^ ILC1 (**B**), CD45^+^FcεR1^−^GATA-3^+^ ILC2 (**D**), and CD45^+^FcεR1^−^RORγt^+^ ILC3 cells (**F**) in the lungs. We present the results as the mean ± SEM (*n* = 6). * *p*  <  0.05 vs. the ND group, # *p* < 0.05 vs. the HFD-treated group.

Because obesity is associated with adipose tissue metaflammation, lung ILC profiles were examined. There are three major types of ILC, ILC1, ILC2, and ILC3, which correspond to Th1, Th2, and Th17 cells, respectively [19]. As shown in Figure 6, HFD significantly increased the CD45^+^FcεR1-T-bet^+^ ILC1, CD45^+^FcεR1-GATA-3^+^ ILC2, and CD45^+^FcεR1-RORγt^+^ ILC3 populations in the lungs, whereas euiin-tang reduced these populations (Figure 6). The presence of numerous macrophages in BALF prompted us to examine the populations of pro-inflammatory F4/80^+^CD86^+^ M1 and anti-inflammatory F4/80^+^CD206^+^ M2 macrophages in the lungs.

Macrophages are immune cells that play a role in inflammation and immunity. Macrophage polarization significantly impacts asthma development. Macrophages can be activated in two ways when recruited to a specific area of the body: classically (M1) or alternatively (M2), depending on the local environment [20]. Obese mice have M1-polarized macrophages [21].

**Figure 7:**
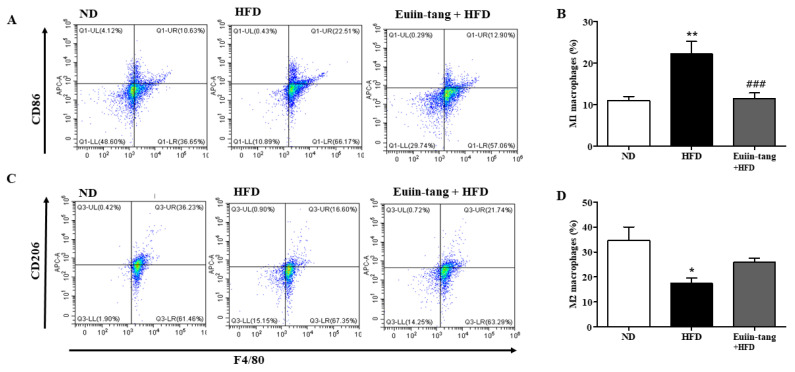
Euiin-tang suppressed the HFD-induced increase in the number of M1 macrophages and the decrease in M2 macrophages in the lungs: Representative flow cytometry dot plots of FACS analysis of F4/80^+^CD86^+^ M1 (**A**) and F4/80^+^CD206^+^ M2 macrophages (**C**) in the lungs. Percentage of F4/80^+^CD86^+^ M1 (**B**) and F4/80^+^CD206^+^ M2 macrophages (**D**) in the lungs. We present the results as the mean ± SEM (*n* = 6). ** *p* < 0.01 and * *p*  <  0.05 vs. the ND group, ### *p* < 0.001 vs. the HFD-treated group.

Figure 7A,B shows that HFD increased M1 macrophages, but not significantly, whereas euiin-tang significantly decreased the number of M1 macrophages. HFD decreased the number of M2 macrophages, and euiin-tang significantly reversed this decrease (Figure 7C,D).

### 2.6. Euiin-Tang Increased Adiponectin Levels and Suppressed Leptin Levels in the Lungs and Gonadal White Adipose Tissue of Mice

**Figure 8:**
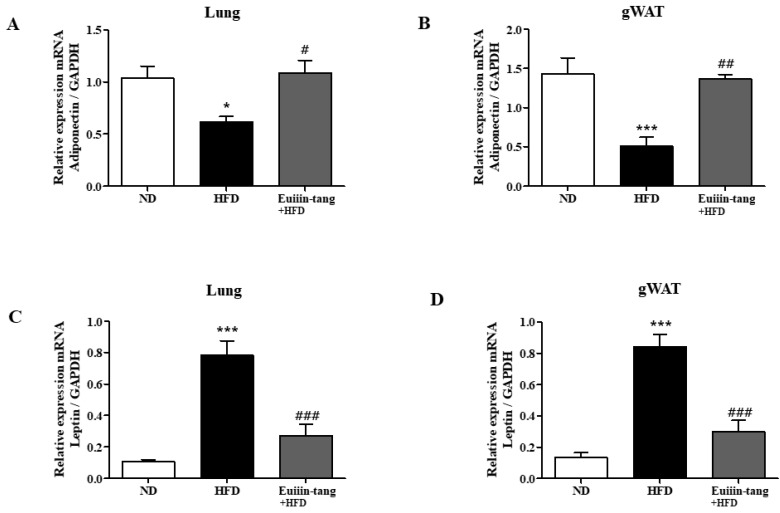
Euiin-tang reversed the HFD-induced changes in adipokine levels in the gonadal adipose tissue and lungs of mice mRNA expression of adiponectin (**A**,**B**) and Leptin (**C**,**D**) in the lungs (**A**,**C**) and gonadal white adipose tissue (**B**,**D**) of mice. The mRNA levels of adipokines are presented as relative expression to GAPDH mRNA levels. We present values as the means ± SEMs (*n* = 6) *** *p* < 0.001 and * *p* < 0.05 vs. the ND group, ## *p* < 0.01, # *p* < 0.05 and ### *p* < 0.001 vs. the HFD-treated group.

Obesity may contribute to OIA by increasing pro-inflammatory adipokine levels. Increased leptin expression has also been linked to obesity-asthma [22]. Adiponectin, another potent anti-inflammatory molecule, is highly expressed strongly in adipocytes. Because obesity regulates the levels of adipokines such as adiponectin and leptin, qRT-PCR was used to analyze the levels of these adipokines in both the lungs and gonadal white adipose tissue. Adiponectin gene levels were significantly decreased in the adipose tissue and lungs of HFD group mice, but euiin-tang significantly reversed this decline (Figure 8A,B). Among the HFD group mice, the levels of the leptin gene significantly increased in adipose tissue and lungs, whereas euiin-tang significantly reduced these levels (Figure 8C,D).

### 2.7. Euiin-Tang Suppressed Inflammatory Cytokine Levels in the BALF of Mice

**Figure 9:**
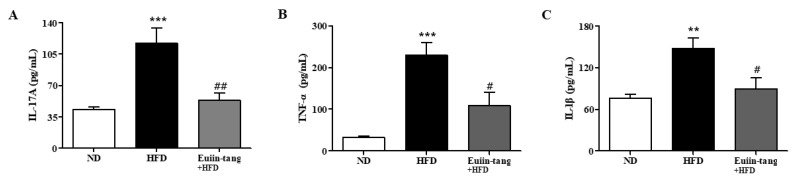
Euiin-tang suppressed the HFD-induced rise in pro-inflammatory cytokines in the BALF of mice: Protein expression of IL-1β (**A**), IL-17A (**B**), and TNF-α (**C**) in the BALF of mice. Protein levels were quantified using ELISA. Values represent the means ± SEMs (*n* = 6) *** *p* < 0.001, ** *p* < 0.01 vs. the ND group. # *p* < 0.05, ## *p* < 0.01 vs. the HFD-treated group.

To confirm the changes in pro-inflammatory cytokine levels, we measured the protein levels of IL-1β, IL-17A, and TNF-α in the BALF. In mice fed an HFD, pro-inflammatory genes were significantly upregulated in the BALF (Figure 9), whereas euiin-tang suppressed this upregulation (Figure 9).

## 3. Discussion

In this study, euiin-tang administration ameliorated HFD-induced obesity and asthma by suppressing metaflammation. Coicis semen, the dried and mature seeds of *Coix lacryma-jobi* L, is the principal component of euiin-tang. According to traditional Chinese medicine, Coicis semen strengthens the spleen and lung functions, reduces dampness, and disperses phlegm [23]. Coicis semen extracts (CSE) have many beneficial effects, including anti-obesity, anti-diabetes, anti-hypertension, and anti-inflammatory properties [24]. Treatment with Coicis semen formula lowers total body fat and subcutaneous fat thickness in addition to reducing inflammation and regulating glucose levels [25,26]. In a db/db diabetic mouse model, Coicis semen exhibited hypoglycemic activity [27]. In obese rats fed an HFD, CSE administration reduced body weight, adipose tissue mass, and food intake [26]. The reductive effects of CSE on body weight, adipose tissue mass, and food intake may have contributed to the suppressive effects of euiin-tang on body and liver weights in the present study. However, euiin-tang did not reduce adiposity, suggesting that CSE and euiin-tang have different mechanisms of action. Neuroendocrine modulation of the brain, as well as decreased expression levels of adipogenesis factors such as fatty acid synthase, peroxisome proliferator-activated receptor γ, and sterol regulatory element binding protein-1c, has been proposed as a CSE mechanism [28,29]. Combinations of Coicis semen and Ephedrae herba have been used to treat obesity in traditional Korean medicine [30,31] because euiin-tang contains both medicinal plants. The combination significantly decreased intracellular lipid accumulation by phosphorylating acetyl-CoA carboxylase, AMP-activated protein kinase, and protein kinase B in 3T3-L1 adipocytes [32]. However, because euiin-tang did not modulate adiposity, we did not examine its neuroendocrine modulation or adipogenesis. The present study focused on HFD-induced metaflammation in adipose tissue. Euiin-tang suppressed HFD-induced metaflammation in adipose tissue, which was supported by decreased levels of pro-inflammatory cytokines in the tissue. The changes in inflammatory cytokine levels in the lungs caused by HFD and Euiin-tang were similar to those in adipose tissues. Additionally, euiin-tang reduced the number of ILC1, ILC2, and ILC3 cells and the ratio of M1/M2 macrophages induced by HFD. Metaflammation and immune responses in the lungs and adipose tissues of C57BL/6 mice can be extrapolated to obesity and OIA in humans. Obesity has been reported to induce significant macrophage infiltration into adipose tissues [33,34]. M1 macrophages typically produce inflammatory responses by releasing TNF-α and IFN-γ, which leads to the activation of NF-κB and the production of NLRP3 and subsequent activation of cytokines such as IL-1β and IL-18 [5]. Activation of the NLRP3 inflammasome can result in excessive inflammation and tissue damage, contributing to chronic inflammatory diseases, such as asthma [35]. Activation of NLRP3 inflammasome-related gene expression with IL-1β exaggerates the asthmatic episode in patients with obesity and asthma [36]. In addition, IL-6 is highly expressed in adult adipose tissue and is positively correlated with obesity [37]. As each ILC plays a unique role in the respiratory system—ILC1 in chronic obstructive pulmonary disease, ILC2 in allergic asthma, and ILC3 in neutrophilic asthma [19]—OIA seems to be a unique disease that also recruits the three ILC cells. HFD increased pro-inflammatory cytokines in Th1, Th2, and Th17 cells, whereas euiin-tang suppressed them. Further investigation is necessary to determine how human obesity and OIA are influenced by ILC1, ILC2, and ILC3.

The presence of adipokines in metaflammation is not commonly observed in other inflammatory diseases such as rheumatoid arthritis, atopic dermatitis, and allergic asthma. Adipokines influence the production of pro-inflammatory and anti-inflammatory cytokines. Leptin, a pro-inflammatory adipokine, stimulates the production of CCL2 in human hepatic stellate cells and activates macrophages to produce pro-inflammatory IL-6, IL-12, and TNF-α [38,39,40]. Patients with obesity and asthma have higher leptin levels than non-obese populations [41]. Asthma severity may be indicated by low adiponectin levels and high body mass index in children [42]. Consequently, the increased leptin and decreased adiponectin levels in HFD-fed mice and their reversal by euiin-tang suggest that modulating adipokines may also be crucial for treating obesity and OIA.

To exclude the influence of sex hormones, only male C57BL/6 mice were used. In obese female asthmatics, adiponectin levels are significantly lower than those in males, implying that both sexes respond differently to adiponectin [43]. Future studies should include female mice to compensate for these limitations.

The gut microbiota has been linked to obesity in recent years [44]. Several studies have linked obesity to an increased Firmicutes/Bacteroidetes ratio in humans [45]. An intervention involving a Coicis seed-based prebiotic and probiotic (*Lactobacillus paracasei* and *Bacillus coagulans*) improved HFD-induced dysbiosis, metabolic disorders, and obesity [46]. Coicis semen acts as a prebiotic that prevents diseases associated with obesity [47]. Coicis semen contains compounds with both insulin-like effects and insulin sensitizers [48]. A limitation of this study is that we did not investigate the gut microbiota and active phytochemicals, although coixans and glycans may be the active constituents of euiin-tang that are responsible for its anti-obesity effects [49,50].

## 4. Materials and Methods

### 4.1. Materials

We purchased euiin-tang from Tsumura & Co. (Tokyo, Japan). Euiin-tang consists of seven herbs, semen of *Coix lacryma-jobi* L. (8 g), radix of *Angelica gigantis* Nakai (4 g), rhizoma of *Atractylodes japonica* Koidz. ex Kitam. (4 g), *Ephedra sinica* Stapf (4 g), cortex of *Cinnamomum verum* J. Presl (3 g), *Paeonia lactiflora* Pall. (3 g), and *Glycyrrhiza uralensis* Fisch. (2 g). Tsumura & Co., has conducted quality control of euiin-tang by measuring active constituents such as β-eudesmol for *Coix lacryma-jobi*, *l*-ephedrine for *Ephedra sinica*, paeoniflorin for *Paeonia lactiflora*, and glycyrrhizic acid for *Glycyrrhiza uralensis* based on techniques of TLC and HPLC (Tsumura & Co.). A rodent diet containing 60 kcal% fat (cat. D12492) was purchased from Research Diets (New Brunswick, NJ, USA). All other chemicals were purchased from Sigma-Aldrich (St. Louis, MO, USA).

### 4.2. Mouse Strain

Male mice of the C57BL/6 strain were provided by Daehan Biolink Korea (Seoul, Republic of Korea). A laboratory animal facility at Kyung Hee University houses mice and provides ad libitum chow and water. The mice were kept in standard plastic cages with sawdust bedding in cages of two per cage, with a controlled environment, which consisted of temperatures ranging 22 °C and 24 °C, humidity of 60 ± 5%, and alternating light/dark cycles (lights were on between 7:00 a.m. and 7:00 p.m.). A review of the protocol with respect to ethics and procedures was conducted by Kyung Hee University’s Institutional Animal Care Committee (KHSASP-23-507).

### 4.3. High-Fat Diet (HFD) Feeding

Mice aged six weeks were randomly divided into three groups. A control group of six C57BL/6 mice (ND) were fed a normal chow diet for fifteen weeks, whereas a group of six HFD mice (HFD, Research Diets, New Brunswick, NJ, USA) were fed a synthetic diet containing 60% (*w*/*w*) fat (Figure 1A). During the last 5 weeks, mice in the HFD + euiin-tang group received water-dissolved euiin-tang (5 mg/10 g, oral administration) per day (*n* = 6, Figure 1A). The dose of 5 mg/10 g was decided based on previous publications [12,13]. Body weights of mice were measured every week, and weights of livers and gonadal adipose tissues were measured after sacrifice.

### 4.4. Oral Glucose Tolerance Test

Prior to the oral glucose tolerance test, the mice were fasted for six hours. An oral dose of two grams of D-(+)-glucose per kilogram of body weight was administered. A baseline glucose level, as well as levels at 0, 15, 30, 60, and 120 min, was measured in tail blood using Barozen II glucometers and strips (Handok, Seoul, Republic of Korea).

### 4.5. Measuring Airway Hyperresponsiveness (AHR) to Methacholine

In order to measure AHR, non-invasive lung function measurements were performed using the PLY-UNR-MS2 (EMKA Technologies, Paris, France). According to the manufacturer’s protocol, the mice were placed in a barometric plethysmographic chamber for 3 min and the enhanced pause (Penh) was measured. Results are expressed as the percentage increase in Penh as a result of methacholine challenges (0, 6.25, 12.5, 25, and 50 mg/mL) [51].

### 4.6. Bronchoalveolar Lavage Fluid (BALF) Cell Counting and Analysis

Immune cells were harvested from the BALF in 0.4 mL of phosphate-buffered saline (PBS). The mixed 40 μL of Wright’s solution and this sample were then loaded into the hemocytometer after mixing 20 μL each. The total number of cells was calculated by multiplying the count of cells. The BALF cells in 50 μL of cell suspension were adhered to glass slides using Cellspin**^®^** centrifuge (Hanil Electric, Seoul, Republic of Korea), fixed in MeOH for 30 s, and stained with May–Grünwald solution for 8 min, followed by Giemsa solution for 12 min. Cells were classified as neutrophils, eosinophils, lymphocytes, or macrophages based on their staining and morphological characteristics. Percentages of each cell type were calculated after counting 200 cells in each instance.

### 4.7. Histological Examination of the Lung

In order to analyze immune cell infiltration, mucus-producing cells, and fibrosis in the lungs, lung tissue sections were stained with hematoxylin and eosin (H&E), periodic acid-Schiff (PAS), and Masson’s trichrome (MT) [51].

### 4.8. Quantitative Reverse Transcription–Polymerase Chain Reaction (qRT-PCR)

The total RNA was isolated from the lungs and gonadal white adipose tissue with Trizol**^®^** (Invitrogen, Waltham, MA, USA). MMLV reverse transcription (Promega, Madison, WI) was used to reverse transcribe RNA into cDNA. Bio-Rad’s CFX Connect Real-Time System (Hercules, CA, USA) was used for qRT-PCR with Thunderbird Next SYBR qPCR Mix. Specific primers of IL-1β (sense 5′-CAGGCAGGCAGTATCACTCA-3′, antisense 5′-TGTCCTCATCCTGGAAGGTC-3′), IL-17A (sense 5′-AAAGCTCAGCGTGTCCAAAC-3′, antisense 5′-ACGTGGAACGGTTGAGGTAG-3′), TNF-α (sense 5′-ACGGCATGGATCTCAAAGAC-3′, antisense 5′-AGATAGCAAATCGGCTGACG-3′), IL-13 (sense 5′-CAGCATGGTATGGAGTGTGG-3′, antisense 5′-AGGCCATGCAATATCCTCTG-3′), IL-6 (sense 5′-CTGATGCTGGTGACAACCAC-3′, antisense 5′-TCCACGATTACCCAGAGAAC-3′), MPO (sense 5′-CACTGGACACTGCAACAACA-3′, antisense 5′-CCATTGCGATTGACTCCAGG-3′), IFN-γ (sense 5′-CGCTACACACTGCATCTTGG-3′, antisense 5′-TTTCAATGACTGTGCCGTGG-3′), NLRP3 (sense 5′-ATGCTGCTTCGACATCTCCT-3′, antisense 5′-GTTTCTGGAGGTTGCAGAGC-3′), CCL2 (sense 5′-TGAATGTGAAGTTGACCCGT-3′, antisense 5′-ACAGAAGTGCTTGAGGTGGT-3′), Leptin (sense 5′-TTCCTGTGGCTTTGGTCCTA-3′, antisense 5′-CGACTGCGTGTGTGAAATGT-3′), Adiponectin (sense 5′-TACTGCAACATTCCGGGACT-3′, antisense 5′-GTAGGTGAAGAGAACGGCCT-3′), and GAPDH (sense 5′-AACTTTGGCATTGTGGAAGG-3′, antisense 5′-GGATGCAGGGATGATGTTCT-3′) were used.

### 4.9. Flow Cytometry

Individual cells from the lungs were stained with the fluorescein isothiocyanate (FITC)-labeled Armenian hamster antibody anti-FcεR1 (cat. 11-5898-81, eBioscience, San Diego, CA, USA), and eFluor 450-labeled rat antibody against CD45 (cat. 48-0451-80, eBioscience) for 45 min at 4 °C to measure innate lymphoid cells, which include group 2 ILC (CD45^+^FcεR1^−^GATA-3^+^), 1 ILC (CD45^+^FcεR1^−^T-bet^+^), and group 3 (CD45^+^FcεR1^−^RORγt^+^). The permeabilized cells were stained at 22 °C for 1 h with rat anti-GATA-3 (cat. 50-9966-41, eBioscience), eFluor 660-labeled mouse anti-T-bet (cat. 50-5825-82, eBioscience), or APC-labeled rat anti-RORγt (cat. 17-6988-82, eBioscience), after fixation with IC fixation buffer (cat. 00-8222-49, eBioscience) at 22 °C for 1 h. To analyze single macrophages from the lungs, we stained them with FITC-labeled rat antibodies against F4/80 (cat. 11-4801-81, eBioscience), and anti-CD86 antibodies (cat. 17-0862-81) or anti-CD206 antibodies (cat. 17-2061-82, eBioscience) at 4 °C for 45 min, following fixation with IC fixation buffer (cat. 00-8222-49, eBioscience) at 22 °C for 1 h. The sorting of cells was performed with a CytoFLEX flow cytometer from Beckman Coulter (Brea, CA, USA).

### 4.10. Enzyme-Linked Immunosorbent Assay (ELISA)

ELISA kits were used to measure IL-17A, TNF-α, and IL-1β levels in BALF from mice. The antibodies used to capture IL-17A (catalog no. 88-7371-88), TNF-α (catalog no. 88-7324-88), and IL-1β (catalog no. BMS6002TWO) were obtained from eBioscience (San Diego, CA, USA). The absorbance was measured at 450 nm using avidin-horseradish peroxidase.

### 4.11. Statistics

In all analyses, data are presented as mean ± standard error of the mean (SEM, *n* = 6). Using GraphPad Prism version 5 software, the significance of the results was determined. Statistical significance was set at ** *p* < 0.01, * *p* < 0.05, and *** *p* < 0.001 vs. the ND group, ## *p* < 0.01, # *p* < 0.05, and ### *p* < 0.001 vs. the HFD group.

## 5. Conclusions

HFD-induced obese C57BL/6 mice developed OIA, and euiin-tang administration suppressed metaflammation in adipose tissue and immune responses in the lungs, resulting in the treatment of obesity-induced asthma. This study suggests a therapeutic application of euiin-tang in OIA.

## Data Availability

Data are available on request.
